# From Amputation to Persistent Pain: A Review of Molecular and Cellular Processes in Phantom Limb Pain

**DOI:** 10.3390/ijms27052107

**Published:** 2026-02-24

**Authors:** Catalin-Bogdan Satala, Andreea Onofrei (Popa), Oana Vrînceanu, Daniela Mihalache

**Affiliations:** 1 “Dunărea de Jos” University of Galati, Faculty of Medicine and Pharmacy, Medical and Pharmaceutical Research Center, 800008 Galati, Romania; andreea.onofrei@ugal.ro (A.O.), daniela.mihalache@ugal.ro (D.M.); 2 Department of Pathology, Clinical County Emergency Hospital, 810325 Brăila, Romania; 3 Doctoral School, “George Emil Palade” University of Medicine, Pharmacy, Science and Technology of Targu Mures, 540136 Targu Mures, Romania; oana.mosora@umfst.ro

**Keywords:** phantom limb pain, limb amputation, neuropathic pain, peripheral nerve injury, spinal cord plasticity, supraspinal networks, neuroinflammation, epigenetic regulation

## Abstract

Phantom limb pain (PLP) is a frequent and often persistent consequence of limb amputation, characterized by pain perceived in the absent limb. Despite decades of research, its biological basis remains incompletely understood, and available treatments often provide inconsistent relief. This reflects the complex and heterogeneous nature of phantom limb pain, which cannot be explained by a single anatomical site or pathological process. Current evidence suggests that phantom limb pain emerges from the interaction of changes occurring at multiple levels of the nervous system. Peripheral nerve injury associated with amputation induces molecular and cellular alterations that may influence early nociceptive signaling. These changes can interact with adaptive and maladaptive responses within the spinal cord, including altered synaptic transmission and neuron–glia interactions, which may facilitate sustained amplification of pain-related signals. At supraspinal levels, long-term adaptations within distributed neural networks involved in sensory, motor, and affective processing may contribute to the persistence of pain perceptions in the absence of ongoing peripheral input. Immune-related signaling and long-term regulation of gene expression further modulate these processes and may contribute to inter-individual variability. In this narrative review, we synthesize current experimental and clinical evidence addressing the molecular and cellular processes associated with phantom limb pain following lower limb amputation. Findings are integrated across peripheral, spinal, and supraspinal levels, with consideration of immune-related and regulatory influences. By highlighting areas of convergence, uncertainty, and existing knowledge gaps, this review aims to provide a coherent biological framework that may support future experimental and translational research in this challenging field.

## 1. Introduction

Phantom limb pain (PLP) is a common and often persistent complication following limb amputation, defined as the perception of pain in the absent limb [1,2,3]. Reported prevalence rates vary widely, reflecting differences in study design and patient populations, yet PLP remains a major contributor to long-term morbidity among amputees. Beyond its clinical impact, PLP represents a compelling biological phenomenon, as it challenges conventional models of nociception by demonstrating that pain can be sustained in the absence of ongoing peripheral sensory input [4,5].

Early conceptual models of PLP emphasized maladaptive cortical reorganization resulting from deafferentation. Seminal neuroimaging and electrophysiological studies demonstrated alterations in somatosensory and motor cortical representations following amputation, supporting the idea that cortical plasticity contributes to phantom sensations and pain [6,7,8]. Although this framework remains influential, it has become increasingly evident that cortical reorganization alone does not fully explain the onset, persistence, or variability of PLP [7,8]. Notably, cortical changes are neither universal among amputees nor consistently correlated with pain intensity, suggesting the involvement of additional mechanisms [8].

Accumulating evidence indicates that PLP arises from complex, multilevel interactions within the nervous system that extend from the periphery to supraspinal structures. Amputation induces profound molecular and cellular changes in injured peripheral nerves, including altered ion channel expression, neurotrophic signaling, and inflammatory responses [9]. These peripheral processes can generate aberrant afferent activity capable of driving spinal cord sensitization. At the spinal level, changes in synaptic transmission, inhibitory control, and glial activation contribute to a pro-nociceptive environment that facilitates the amplification and persistence of pain signals. Such mechanisms are well-documented in neuropathic pain models and are increasingly considered relevant to PLP [10].

At supraspinal levels, sustained alterations in neural circuits involved in sensory integration, motor control, and affective processing further shape the experience of phantom pain. Beyond gross structural reorganization, recent studies point toward molecular adaptations within these networks, including activity-dependent signaling pathways and long-term regulation of gene expression. These molecular changes may contribute to the stabilization of maladaptive neural representations, supporting the concept of a persistent “pain memory” even after the loss of peripheral input [11,12].

Neuroimmune interactions have also emerged as important modulators of pain chronification following nerve injury [13]. Peripheral immune activation, spinal glial responses, and central neuroinflammatory signaling have been shown to influence neuronal excitability and synaptic plasticity through the release of cytokines, chemokines, and growth factors [14]. While the role of neuroinflammation in chronic neuropathic pain is increasingly recognized, its specific contribution to PLP remains incompletely characterized and warrants focused synthesis [15].

Despite extensive research into individual components of PLP pathophysiology, the molecular mechanisms underlying this condition are often discussed in a fragmented manner. Many studies address peripheral, spinal, or supraspinal processes in isolation, and findings from general neuropathic pain models are frequently extrapolated to PLP without comprehensive integration [16]. This lack of a unified molecular framework limits mechanistic insight and hinders the formulation of testable hypotheses specific to PLP, particularly in the context of lower limb amputation [15,16]. Throughout this review, we explicitly distinguish between findings directly demonstrated in clinical or experimental studies of PLP and mechanistic insights extrapolated from broader neuropathic pain models. While such extrapolations provide biologically plausible frameworks, they should not be interpreted as definitive evidence specific to PLP.

The aim of this narrative review is to synthesize current evidence on the molecular and cellular mechanisms underlying PLP following lower limb amputation. By integrating data from peripheral nerve remodeling, spinal cord sensitization, supraspinal molecular plasticity, and neuroimmune signaling, this review seeks to provide a coherent mechanistic perspective and to highlight key knowledge gaps that may guide future experimental and translational research.

## 2. Materials and Methods

### 2.1. Literature Search Strategy

A narrative review of the literature was conducted to identify experimental and clinical studies addressing the molecular and cellular mechanisms underlying phantom limb pain following lower limb amputation. The literature search was performed using the PubMed/MEDLINE, Web of Science, and Scopus databases, selected to ensure comprehensive coverage of biomedical, molecular, and translational research. Search strategies combined controlled vocabulary terms and free-text keywords related to phantom limb pain and mechanistic pain research. Search terms included combinations of “phantom limb pain”, “amputation”, “neuropathic pain”, “peripheral nerve injury”, “central sensitization”, “molecular mechanisms”, “ion channels”, “neuroinflammation”, “microglia”, “neurotrophic factors”, and “epigenetic regulation”. Boolean operators were used to refine and expand searches as appropriate. The literature search primarily focused on articles published in English from the early 1990s to the present, reflecting the period during which molecular and cellular approaches to pain research have been most extensively developed. Earlier seminal publications were included where necessary to provide historical and conceptual context.

### 2.2. Study Selection and Eligibility Criteria

Original research articles, systematic reviews, and high-quality narrative reviews were considered eligible for inclusion. Priority was given to studies that provided mechanistic insights at the molecular or cellular level, including investigations of peripheral nerve remodeling, spinal cord sensitization, supraspinal plasticity, neuroimmune signaling, and epigenetic regulation relevant to chronic pain. Both preclinical studies (including in vitro systems and animal models of nerve injury or amputation) and clinical studies involving amputee populations were included when they addressed molecular or cellular aspects of pain mechanisms. Clinical trials were considered when they offered mechanistic or translational insights relevant to PLP or neuropathic pain pathways. Studies focusing exclusively on functional outcomes, rehabilitation strategies, or psychological aspects of PLP without mechanistic relevance were not prioritized. Case reports and small observational studies were included selectively when they provided unique mechanistic or translational perspectives.

### 2.3. Identification of Key Publications

To identify seminal studies and highly influential publications, citation tracking and reference list screening were performed for key articles identified through database searches. Foundational studies describing cortical reorganization following amputation, molecular changes in peripheral nerve injury, and spinal cord mechanisms of central sensitization were considered essential contributions and were included regardless of publication date. More recent studies were selected based on their impact on understanding specific molecular pathways, such as ion channel dysregulation, glutamatergic signaling, glial activation, neuroimmune interactions, and epigenetic mechanisms. Where possible, findings replicated across independent studies and experimental models were prioritized over isolated reports.

### 2.4. Clinical Trials and Translational Evidence

Clinical trials related to phantom limb pain were reviewed primarily for their mechanistic implications rather than for therapeutic efficacy alone. Trials investigating pharmacological agents, neuromodulatory approaches, or interventions targeting neural plasticity were included when they provided insight into underlying molecular or cellular pathways. Given the limited number of trials specifically designed to investigate molecular mechanisms in phantom limb pain, studies conducted in broader neuropathic pain populations were also considered when their findings were biologically relevant to post-amputation pain. Key clinical trials are discussed in the context of their mechanistic contributions, and selected trials may be summarized in tabular form for clarity.

### 2.5. Data Extraction and Narrative Synthesis

Relevant data were extracted manually from selected publications, focusing on molecular targets, signaling pathways, cell types involved, and experimental or clinical context. Due to the heterogeneity of study designs, models, and outcome measures, a quantitative meta-analysis was not performed. Instead, findings were synthesized using a narrative approach, integrating evidence across different levels of the nervous system and experimental paradigms. The literature was organized thematically into sections addressing peripheral, spinal, and supraspinal molecular mechanisms, as well as neuroimmune and epigenetic contributions to phantom limb pain. Based on these aspects, the initial database search yielded approximately 880 records. After removal of duplicates and screening of titles and abstracts for relevance to molecular and cellular mechanisms of PLP or related neuropathic processes, approximately 230 articles were assessed in full text. From these, studies were selectively integrated based on mechanistic depth, methodological rigor, and relevance to post-amputation pain. The final reference list reflects the most conceptually relevant publications and does not represent an exhaustive enumeration of all screened studies.

### 2.6. Methodological Considerations

As a narrative review, the present study was designed to provide a structured and integrative synthesis of the available literature rather than a quantitative systematic analysis. The objective was to organize mechanistic evidence across peripheral, spinal, and supraspinal levels within a coherent conceptual framework. Given the heterogeneity of study designs, experimental models, and clinical methodologies in the field, a thematic narrative approach was considered most appropriate for integrating findings from basic science and translational research. Study selection was guided by mechanistic relevance, conceptual contribution, and methodological rigor, with priority given to publications that informed biological understanding of PLP. This methodological approach allows flexibility in synthesizing diverse forms of evidence while emphasizing biological plausibility and cross-level integration.

### 2.7. Study Limitations

Several limitations constrain current understanding of PLP at the molecular and cellular level. First, a substantial proportion of available mechanistic evidence is derived from animal models of neuropathic pain rather than directly from amputee populations. While such models provide valuable biological insight, they may not fully capture the complexity of human PLP. Second, direct molecular investigations in human spinal and supraspinal tissues are inherently limited due to ethical and technical constraints. Consequently, many supraspinal mechanisms discussed in this review rely on indirect evidence from neuroimaging or translational inference. Third, heterogeneity in patient characteristics, including amputation etiology, time since amputation, comorbid conditions, and psychological factors, complicates interpretation of mechanistic findings and limits generalizability. Finally, the temporal relationship between early post-amputation biological changes and long-term pain persistence remains insufficiently characterized. These limitations emphasize that current mechanistic models of PLP should be regarded as provisional and hypothesis-generating rather than definitive.

## 3. Peripheral Molecular Alterations Associated with Amputation-Related Nerve Injury

Lower limb amputation inevitably involves transection of peripheral nerves, a process known to initiate a broad range of molecular and cellular responses within sensory neurons [17]. A substantial body of experimental literature has demonstrated that peripheral nerve injury induces long-lasting changes in gene and protein expression that modify neuronal excitability and synaptic signaling [18]. Although phantom limb pain is not solely determined by peripheral mechanisms, such alterations are widely considered to contribute to the initiation of maladaptive nociceptive processing [19].

Among the most consistently reported molecular changes following peripheral nerve injury is the altered expression and distribution of voltage-gated sodium channels in primary afferent neurons [20]. Preclinical studies and investigations in human neuropathic pain conditions have documented increased expression of *Nav1.7*, *Nav1.8*, *and Nav1.9* subtypes in injured sensory neurons [21]. These changes are associated with enhanced membrane excitability and spontaneous ectopic discharges, which are thought to represent a source of abnormal afferent input [22]. Direct evidence linking these alterations specifically to PLP populations is currently scarce, and most available data derive from broader neuropathic pain research. Therefore, their relevance to PLP should be interpreted as biologically plausible but not yet conclusively demonstrated in amputee cohorts [9].

In addition to sodium channels, changes in calcium channel activity have been observed following nerve injury and deafferentation [23]. Enhanced calcium influx in primary sensory neurons has been shown to facilitate neurotransmitter release at central terminals, thereby strengthening synaptic transmission in the dorsal horn [24]. Such mechanisms have been primarily characterized in experimental neuropathic pain models and have not been directly demonstrated in PLP-specific investigations. Their applicability to post-amputation pain remains inferential and requires direct validation in amputee populations [18].

Transient receptor potential (TRP) channels have been implicated in peripheral nociceptor sensitization in response to inflammatory and injury-related signals [25]. Experimental studies indicate that channels such as *TRPV1* and *TRPA1* can be upregulated or functionally sensitized following peripheral nerve injury, particularly in inflammatory microenvironments [26]. These channels contribute to stimulus-evoked and spontaneous nociceptor activity, although their specific contribution to PLP has not been conclusively established [27].

Neurotrophic factors, including nerve growth factor and brain-derived neurotrophic factor, are known to be upregulated following nerve injury and to influence neuronal survival, axonal sprouting, and synaptic plasticity [28]. Sustained neurotrophin signaling has been associated with increased nociceptor excitability and altered ion channel expression in neuropathic pain models [29]. In the context of amputation, such molecular changes may modulate peripheral inputs that interact with central pain-processing circuits, though their direct role in maintaining PLP remains to be fully elucidated [30].

Neuroma formation at the site of nerve transection represents a distinctive anatomical and molecular feature of amputation [31]. Studies examining neuromas have reported disorganized axonal growth, altered ion channel clustering, and increased expression of inflammatory mediators [32]. These characteristics have been associated with spontaneous and mechanically induced ectopic activity in injured nerves [9]. While neuromas are more commonly linked to residual limb pain, their contribution to PLP is less clear and likely variable among individuals [33].

Peripheral immune responses further shape the molecular environment following nerve injury. Infiltration of immune cells and the release of cytokines and chemokines have been shown to sensitize nociceptors and modulate intracellular signaling pathways within sensory neurons [13,14,34]. These immune-mediated effects are well documented in neuropathic pain models and may influence early nociceptive processing after amputation [18,34]. However, whether peripheral inflammation continues to play a significant role in established PLP remains an open question.

Taken together, available evidence suggests that peripheral molecular alterations following amputation-related nerve injury generate a state of enhanced nociceptor excitability and aberrant afferent signaling.

## 4. Spinal Cord Molecular Mechanisms and Central Sensitization

Peripheral nerve injury associated with limb amputation is known to induce profound and long-lasting changes within the spinal cord, particularly at the level of the dorsal horn [34]. These changes are commonly referred to as central sensitization and are characterized by an increased responsiveness of spinal neurons to both nociceptive and non-nociceptive inputs [35]. Although central sensitization has been most extensively studied in the context of neuropathic pain, a growing body of evidence suggests that similar spinal mechanisms may contribute to the development and persistence of phantom limb pain [19].

One of the hallmark features of spinal sensitization following nerve injury is enhanced excitatory synaptic transmission within the dorsal horn. Experimental studies have demonstrated increased glutamate release from primary afferent terminals and altered postsynaptic responsiveness of dorsal horn neurons [36,37]. At the molecular level, these changes are associated with increased activity and phosphorylation of ionotropic glutamate receptors, particularly NMDA and AMPA receptors [38]. Enhanced NMDA receptor function has been implicated in activity-dependent synaptic plasticity and prolonged excitatory signaling, which may lower the threshold for pain transmission. For example, experimental studies have shown that pharmacological blockade of NMDA receptors in rodent nerve injury models attenuates mechanical allodynia and reduces dorsal horn neuronal hyperexcitability, supporting a causal contribution of NMDA-dependent plasticity to pain amplification [38,39]. While direct evidence linking NMDA receptor modulation specifically to PLP remains limited, the well-established role of these receptors in neuropathic pain provides a plausible mechanistic link [39,40].

In parallel with increased excitatory drive, inhibitory control within the spinal cord is often compromised following peripheral nerve injury [41]. Several studies have reported a reduction in GABAergic and glycinergic inhibition in dorsal horn circuits, partly mediated by BDNF-dependent downregulation of the potassium-chloride cotransporter, resulting in an imbalance between excitation and inhibition [29]. This disinhibition may arise from multiple mechanisms, including decreased neurotransmitter release, altered receptor expression, or changes in chloride homeostasis within spinal neurons [42]. Such alterations can amplify nociceptive signaling and facilitate the spread of excitation to normally non-nociceptive pathways. Although the extent to which spinal disinhibition contributes specifically to PLP has not been fully delineated, it is widely regarded as a key component of central sensitization in chronic pain states [35,37].

Glial cells have emerged as critical modulators of spinal cord plasticity following nerve injury [34]. Microglia and astrocytes become activated in response to peripheral nerve damage and contribute to the maintenance of a pro-nociceptive spinal environment [13]. Activated microglia release a range of signaling molecules, including pro-inflammatory cytokines, chemokines, and neurotrophic factors, which can directly modulate neuronal excitability and synaptic transmission. Astrocytes, in turn, play an important role in regulating extracellular glutamate levels and synaptic homeostasis, and their dysregulation may further enhance excitatory signaling [15].

Microglial activation is often considered an early event in spinal sensitization [43]. Experimental models of nerve injury have shown that microglial activation occurs rapidly after axonal damage and precedes the development of sustained pain behaviors [44]. Molecular pathways implicated in microglial activation include purinergic signaling, toll-like receptor pathways, and intracellular kinase cascades [45]. These pathways lead to the release of cytokines such as tumor necrosis factor-alpha and interleukin-1 beta, which have been shown to potentiate excitatory synaptic transmission and reduce inhibitory signaling in dorsal horn neurons [46]. Although most of this evidence derives from animal models of neuropathic pain rather than PLP-specific studies, it provides a mechanistic framework that may be relevant to post-amputation pain conditions. However, direct molecular evidence from human PLP spinal tissue remains extremely limited [19].

Astrocytic responses tend to develop more gradually but are thought to contribute to the maintenance of chronic pain [47]. Reactive astrocytes exhibit altered expression of glutamate transporters and gap junction proteins, which can disrupt synaptic clearance of excitatory neurotransmitters and promote sustained neuronal activation [48]. Additionally, astrocytes can release gliotransmitters and inflammatory mediators that further modulate synaptic plasticity [49]. The relative contribution of astrocytes versus microglia to PLP remains uncertain, and it is likely that their roles vary across different stages of pain chronification [50].

Neuroimmune interactions within the spinal cord represent another important dimension of central sensitization [51]. Peripheral nerve injury leads to increased permeability of the blood–spinal cord barrier and facilitates the recruitment of immune cells, as well as the diffusion of circulating inflammatory mediators [52]. These immune signals can interact with resident glial cells and neurons, reinforcing pro-nociceptive signaling pathways [53]. While the extent of immune cell infiltration in human PLP is not well characterized, indirect evidence supports a role for sustained neuroinflammatory signaling in chronic pain conditions following nerve injury [54].

Intracellular signaling cascades within spinal neurons also play a central role in the development of sensitization [55]. Activation of mitogen-activated protein kinase pathways, including *ERK*, *p38*, *and JNK*, has been widely reported in dorsal horn neurons and glial cells following nerve injury [56]. These kinases regulate transcriptional and post-translational processes that influence receptor expression, ion channel function, and synaptic strength [57]. Such molecular adaptations can stabilize sensitized states within spinal circuits, contributing to the persistence of pain even after the initial injury has healed [58].

Importantly, spinal sensitization is not a static process but evolves over time. Early changes driven by acute injury and inflammation may transition into more stable molecular and epigenetic alterations that sustain altered neural responsiveness [59,60]. Experimental studies suggest that repeated or persistent nociceptive input can induce long-term changes in gene expression within spinal neurons, potentially contributing to a form of molecular memory of pain [61]. Whether similar mechanisms operate in PLP, where peripheral input may be intermittent or absent, remains an area of active investigation [5].

Another aspect of spinal plasticity relevant to PLP is the reorganization of sensory input processing [36]. Following deafferentation, dorsal horn neurons may receive altered patterns of input from adjacent spinal segments or from remaining sensory afferents [62]. Such reorganization has been observed in animal models and may contribute to abnormal sensory perceptions, including pain referred to the missing limb. Although the molecular underpinnings of this process are incompletely understood, changes in synaptic connectivity and receptor expression are likely involved [63,64].

Despite the substantial number of studies supporting a role for spinal sensitization in neuropathic pain, several limitations should be acknowledged when extrapolating these findings to PLP [65]. Many mechanistic studies rely on animal models that do not fully recapitulate the complexity of human amputation and its long-term consequences. Furthermore, direct molecular analyses of spinal cord tissue in individuals with PLP are inherently limited. Consequently, spinal sensitization in PLP should currently be regarded as a strongly supported mechanistic hypothesis rather than a directly demonstrated molecular pathway in amputee-specific cohorts [12,66,67].

Taken together, available evidence indicates that spinal cord molecular and cellular plasticity represents a critical intermediate stage between peripheral nerve injury and supraspinal pain processing [68,69]. Enhanced excitatory transmission, impaired inhibition, glial activation, and neuroimmune signaling collectively contribute to a sensitized spinal environment capable of amplifying and sustaining nociceptive signals.

## 5. Supraspinal Molecular Plasticity and the Concept of Pain Memory

Beyond the spinal cord, limb amputation is associated with long-lasting changes in supraspinal structures involved in sensory perception, motor control, and affective processing [70]. These changes have traditionally been described in terms of large-scale cortical reorganization; however, increasing attention has been directed toward the molecular and cellular mechanisms that underlie such plasticity [64]. While direct molecular evidence in human phantom limb pain remains limited, converging data from neuroimaging, experimental models, and neuropathic pain research suggest that supraspinal molecular adaptations may contribute to the persistence of phantom pain [30].

It is important to note that most molecular-level evidence in this domain originates from neuropathic pain research more broadly, whereas human PLP investigations have predominantly relied on neuroimaging and behavioral assessments. Direct molecular characterization of supraspinal tissue in PLP remains scarce.

Early studies of PLP emphasized alterations in the primary somatosensory and motor cortices following deafferentation [71]. Functional and structural imaging studies demonstrated shifts in cortical representations corresponding to the missing limb, which were initially interpreted as maladaptive plasticity [72]. In one of the seminal studies, Flor et al. reported that the extent of somatosensory cortical reorganization correlated with phantom pain intensity in upper limb amputees, whereas later studies failed to replicate this association consistently [40]. Although these findings provided an important conceptual framework, subsequent research revealed considerable inter-individual variability and inconsistent correlations between the extent of cortical reorganization and pain intensity. These observations indicate that cortical reorganization alone is insufficient to explain the complexity of PLP and suggest the involvement of additional mechanisms. Moreover, some authors have questioned whether cortical remapping should be interpreted as inherently maladaptive, proposing that certain forms of reorganization may reflect adaptive compensatory processes. The inconsistent correlation between cortical changes and pain intensity further complicates causal interpretation and suggests that cortical alterations may represent correlates rather than primary drivers of PLP [73,74].

At a molecular level, cortical and subcortical plasticity is known to be regulated by activity-dependent signaling pathways that modulate synaptic strength and connectivity [75]. Neurotrophic factors, particularly brain-derived neurotrophic factor (BDNF), play a central role in synaptic plasticity and experience-dependent remodeling of neural circuits, by long-term potentiation and synaptic strengthening within thalamocortical circuits, processes that may stabilize maladaptive representations following limb loss and contribute to persistent pain states [76,77]. Experimental studies in neuropathic pain models have shown that altered neurotrophic signaling within cortical and thalamic regions can influence pain perception by modulating excitatory and inhibitory synaptic transmission. While direct evidence in PLP is sparse, these mechanisms are biologically plausible given the profound changes in afferent input following amputation [77,78].

Alterations in excitatory–inhibitory balance represent another key aspect of supraspinal plasticity. Chronic pain conditions have been associated with reduced inhibitory neurotransmission and enhanced excitatory signaling within cortical and limbic networks. Molecular studies suggest that changes in receptor expression, neurotransmitter availability, and intracellular signaling cascades contribute to this imbalance. Such alterations may increase the salience of pain-related signals and facilitate their persistence over time. In the context of PLP, similar mechanisms could contribute to the maintenance of pain representations in the absence of peripheral input [79].

The thalamus occupies a central position in pain processing and sensory integration and has been implicated in chronic pain states following peripheral nerve injury. Neuroimaging studies have reported functional and metabolic changes in thalamic nuclei in amputees, suggesting altered thalamocortical communication [80]. At the molecular level, changes in glutamatergic transmission, receptor phosphorylation, and synaptic plasticity within thalamic circuits have been described in experimental models of neuropathic pain. These alterations may influence the relay and modulation of nociceptive signals, although their specific contribution to PLP remains incompletely characterized [81].

Beyond sensory pathways, supraspinal regions involved in affective and cognitive processing, including the anterior cingulate cortex, insular cortex, and limbic structures, have been implicated in the experience of phantom pain. These regions are known to integrate sensory input with emotional and contextual information and are sensitive to chronic pain-related plasticity. Molecular adaptations within these networks, such as altered neurotransmitter systems and stress-related signaling pathways, may modulate pain perception and its emotional valence. However, distinguishing mechanisms specific to PLP from those common to chronic pain more broadly remains challenging [82].

An emerging concept in chronic pain research is that of a persistent “pain memory,” whereby molecular and synaptic changes stabilize maladaptive neural representations over time [65]. This concept does not imply a literal memory trace but rather refers to long-term alterations in gene expression, synaptic architecture, and network connectivity that maintain heightened pain sensitivity. Epigenetic mechanisms, including DNA methylation, histone modifications, and microRNA-mediated regulation, have been proposed as potential contributors to such long-lasting changes. Evidence for epigenetic involvement in neuropathic pain is increasing, but direct data linking these processes to PLP are currently limited [83].

Importantly, supraspinal plasticity should not be viewed as a uniform or static phenomenon. Longitudinal studies suggest that brain changes following amputation may evolve over time and may be influenced by factors such as residual limb pain, prosthesis use, sensory feedback, and psychological state. Molecular mechanisms underlying this dynamic plasticity are likely to be complex and context-dependent, involving interactions between neuronal activity, neuroimmune signaling, and systemic factors. As such, supraspinal contributions to PLP are best understood as part of a broader, distributed network rather than as isolated cortical abnormalities [69].

Most molecular insights are derived from animal models or extrapolated from other chronic pain conditions, and direct access to human supraspinal tissue is inherently limited. Neuroimaging studies provide valuable functional and structural information but cannot directly resolve underlying molecular processes. Consequently, existing models of supraspinal involvement in PLP remain largely inferential and require cautious interpretation [84].

Rather than representing a single locus of dysfunction, supraspinal involvement in PLP appears to reflect a distributed pattern of molecular and cellular adaptations across interconnected brain regions. Activity-dependent signaling pathways, altered neurotransmission, and long-term regulatory mechanisms may collectively influence how pain-related information is processed and maintained following amputation [64]. Importantly, the relative contribution of supraspinal molecular mechanisms is likely to vary across individuals and over time, emphasizing the need for cautious interpretation and for future studies that integrate molecular, neuroimaging, and clinical data [11].

## 6. Neuroimmune and Epigenetic Contributions to Phantom Limb Pain

Increasing attention has been directed toward the role of neuroimmune interactions in the development and persistence of chronic pain following peripheral nerve injury. In the context of limb amputation, immune responses are initiated at multiple levels, including the peripheral nervous system, spinal cord, and supraspinal structures. While the involvement of immune signaling in phantom limb pain has not been fully delineated, a growing body of evidence from neuropathic pain research suggests that neuroimmune mechanisms may modulate neuronal excitability and plasticity over extended time scales. However, most molecular evidence supporting this view derives from non-amputation neuropathic models, and direct validation in PLP-specific populations remains limited [14].

Peripheral nerve injury is accompanied by a robust immune response characterized by the activation and recruitment of innate immune cells. Macrophages, mast cells, and other immune populations release cytokines, chemokines, and growth factors that can influence nociceptor function. These mediators have been shown to alter ion channel activity, receptor sensitivity, and intracellular signaling pathways within sensory neurons. Although such mechanisms have been primarily studied in non-amputation models of neuropathic pain, the molecular consequences of nerve transection suggest potential relevance to early post-amputation nociceptive processing [34].

Within the spinal cord, neuroimmune signaling is largely mediated by glial cells, particularly microglia and astrocytes. Following peripheral nerve injury, microglia undergo phenotypic changes associated with increased responsiveness to neuronal activity and injury-related signals. Activated microglia release a range of soluble mediators that can enhance excitatory synaptic transmission and suppress inhibitory signaling in dorsal horn neurons. For instance, Tsuda et al. demonstrated that inhibition of microglial P2 × 4 receptors attenuated neuropathic pain behaviors in rodents, linking specific microglial signaling pathways to pain hypersensitivity [85]. In addition to cytokines, brain-derived neurotrophic factor (BDNF) released by activated microglia has been shown to downregulate the potassium–chloride cotransporter KCC2 in dorsal horn neurons, thereby altering chloride homeostasis and reducing the efficacy of GABAergic inhibition, a mechanism strongly involved in neuropathic pain hypersensitivity [29]. Astrocytes contribute to this process through alterations in glutamate uptake, metabolic support, and the release of gliotransmitters. These glial responses are well-documented in models of chronic neuropathic pain and are thought to play a role in maintaining central sensitization. Nevertheless, not all neuropathic pain models demonstrate uniform patterns of glial activation, and sex-dependent differences in immune–neural interactions have been reported. Furthermore, reliable biomarkers of sustained neuroinflammation in human PLP populations have not yet been established, underscoring the translational gap between preclinical findings and clinical evidence. Importantly, the magnitude and persistence of glial activation reported across experimental studies vary substantially depending on injury model, species, and methodological approach. Some studies have failed to demonstrate sustained microglial activation beyond the early post-injury phase, raising questions about its long-term role in pain maintenance. Moreover, the relative contribution of immune-mediated versus neuron-intrinsic mechanisms remains debated. These inconsistencies highlight that neuroimmune involvement in PLP should not be viewed as uniform or universally dominant, but rather as context-dependent and potentially heterogeneous across individuals [85].

Importantly, neuroimmune signaling within the spinal cord is not limited to acute injury responses. Sustained glial activation and prolonged cytokine release have been observed in chronic pain models long after the initial insult. Such persistent neuroinflammatory states may contribute to the stabilization of sensitized neural circuits, even in the absence of ongoing peripheral input. In the case of PLP, this raises the possibility that early immune-mediated events following amputation may have long-term consequences for spinal and supraspinal pain processing, although direct evidence in human PLP remains limited [13].

At supraspinal levels, immune-related signaling pathways have also been implicated in chronic pain conditions. Cytokines and chemokines can influence neuronal function within the brain either through local production by resident immune cells or via peripheral-to-central signaling mechanisms. Microglial activation has been reported in several brain regions involved in pain perception and affective processing in experimental models of nerve injury. These findings suggest that neuroimmune mechanisms may extend beyond the spinal cord to shape broader pain-related networks, although their specific role in PLP requires further investigation [86].

In parallel with immune-mediated processes, epigenetic mechanisms have emerged as potential contributors to long-lasting changes in pain-related neural circuits. Epigenetic regulation refers to heritable or stable modifications of gene expression that do not involve changes in DNA sequence, including DNA methylation, histone modifications, and non-coding RNA-mediated regulation. Such mechanisms are well suited to mediate persistent alterations in cellular phenotype in response to transient environmental or injury-related stimuli [60].

Experimental studies have demonstrated that peripheral nerve injury can induce epigenetic changes within sensory neurons, spinal cord cells, and supraspinal structures. These changes have been associated with altered expression of genes involved in neurotransmission, inflammation, and synaptic plasticity. In neuropathic pain models, epigenetic modulation has been shown to influence pain sensitivity and the maintenance of chronic pain states. While analogous data in PLP are scarce, the shared features of nerve injury and long-term plasticity suggest that epigenetic mechanisms may contribute to sustained pain representations following amputation [87].

MicroRNAs have attracted particular interest as regulators of post-injury gene expression. Several microRNAs have been shown to modulate inflammatory signaling, ion channel expression, and synaptic function in models of neuropathic pain. Altered microRNA expression profiles have been reported following nerve injury and are thought to contribute to long-term changes in neuronal and glial function. Whether specific microRNA signatures are associated with PLP remains unknown, but this represents an area of potential relevance for future research [88].

Epigenetic processes may also interact with neuroimmune signaling pathways. Inflammatory mediators can influence epigenetic enzymes and chromatin structure, thereby linking immune activation to long-term transcriptional changes. Conversely, epigenetic regulation can shape immune cell phenotypes and cytokine production. Such bidirectional interactions suggest that neuroimmune and epigenetic mechanisms should not be considered in isolation but rather as components of an integrated regulatory network that influences pain chronification [89].

**Figure 1:**
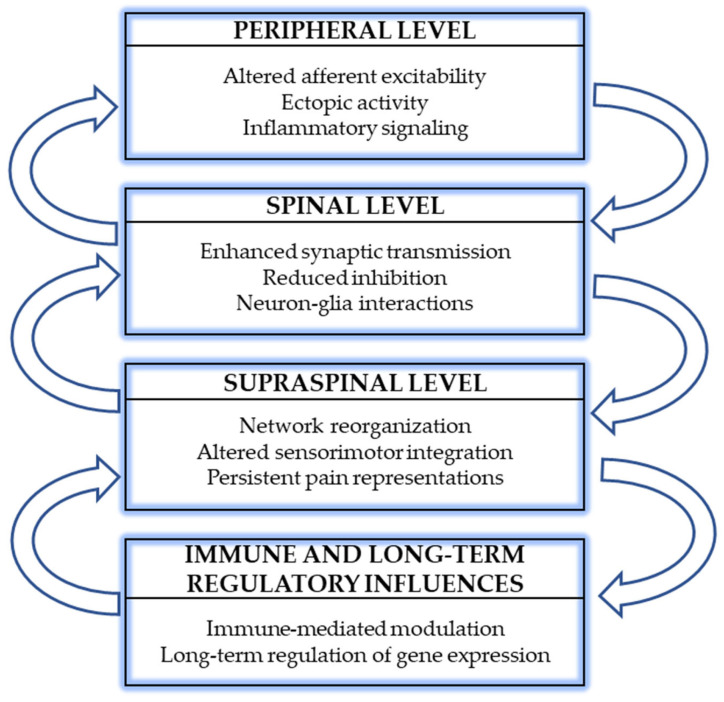
Multilevel biological processes involved in phantom limb pain (PLP): peripheral, spinal and supraspinal processes, together with immune-related and long-term regulatory influences that can interact in both ascending and descending directions.

Rather than acting as primary drivers of PLP, neuroimmune and epigenetic mechanisms may be best viewed as modulatory processes that shape the persistence and intensity of pain-related neural activity. By influencing neuronal excitability, synaptic plasticity, and gene expression across multiple levels of the nervous system, these mechanisms may contribute to the stabilization of maladaptive pain networks following amputation (Figure 1). Further research integrating molecular, cellular, and systems-level approaches will be required to clarify their specific roles and therapeutic relevance [39].

## 7. Integrative Pathophysiological Models and Emerging Hypotheses

The molecular and cellular mechanisms discussed across peripheral, spinal, and supraspinal levels highlight the multifactorial nature of phantom limb pain. Based on currently available evidence, much of which is extrapolated from neuropathic pain research, PLP appears to reflect the convergence of multiple processes initiated by nerve injury and sustained through maladaptive plasticity within distributed neural networks. An integrative framework is therefore required to reconcile findings derived from diverse experimental models and clinical observations. One unifying concept emerging from the literature is that amputation-related nerve injury generates an initial cascade of molecular events at the peripheral level that may prime the central nervous system for long-term sensitization. Altered ion channel expression, neurotrophic signaling, and immune activation in injured nerves can lead to aberrant afferent activity during the early post-amputation period. Although such peripheral inputs may diminish over time, their initial impact on spinal and supraspinal circuits may trigger molecular and synaptic changes that persist independently of ongoing peripheral signaling [18,65].

Within the spinal cord, these early peripheral signals are thought to interact with intrinsic plasticity mechanisms that amplify and stabilize nociceptive transmission. Enhanced excitatory synaptic signaling, impaired inhibitory control, and sustained glial activation collectively contribute to a sensitized dorsal horn environment. Importantly, spinal plasticity is increasingly viewed as a dynamic process that evolves from transient injury-related responses toward more stable molecular adaptations. These adaptations may lower the threshold for pain transmission and facilitate the propagation of aberrant signals to supraspinal structures [35].

At supraspinal levels, deafferentation and altered spinal input are associated with reorganization of pain-related networks rather than isolated cortical changes. Molecular adaptations within these networks, including activity-dependent signaling pathways and long-term regulation of gene expression, may contribute to the persistence of pain-related representations. In this context, PLP can be conceptualized as a disorder of network-level plasticity, in which sensory, motor, and affective components of pain processing become dysregulated through interacting molecular mechanisms. Neuroimmune and epigenetic processes may provide an additional layer of integration across anatomical levels. Immune-derived mediators can influence neuronal excitability and synaptic function at peripheral, spinal, and supraspinal sites, while epigenetic regulation offers a plausible mechanism for maintaining long-lasting changes in gene expression following transient injury-related stimuli. The interaction between immune signaling and epigenetic modulation may be particularly relevant for understanding how early post-amputation events lead to persistent alterations in pain processing [12].

Based on these observations, several mechanistic hypotheses can be proposed. One hypothesis is that PLP reflects the stabilization of a maladaptive pain network initiated by peripheral nerve injury and reinforced through spinal sensitization and supraspinal plasticity. Another possibility is that individual variability in immune responses, neurotrophic signaling, or epigenetic regulation influences susceptibility to PLP and contributes to its heterogeneity. These hypotheses remain speculative but provide a structured framework for future experimental investigation [82].

Much of the molecular data underlying these frameworks is derived from animal models of neuropathic pain, and direct validation in human PLP is limited. Furthermore, the temporal relationships between peripheral injury, central plasticity, and pain perception are not fully resolved. As such, proposed models should be viewed as heuristic tools rather than definitive explanations. Future research efforts may benefit from approaches that explicitly link molecular changes to systems-level outcomes, such as combining molecular profiling with neuroimaging, electrophysiology, and longitudinal clinical assessment. Such integrative strategies may help clarify the relative contributions of different mechanisms across stages of phantom limb pain and identify points of convergence that are most relevant for mechanistic understanding [64].

In this context, conceptual models that integrate peripheral, spinal, supraspinal, neuroimmune, and epigenetic mechanisms offer a useful framework for organizing existing evidence and guiding hypothesis-driven research (Table 1). While these models do not yet provide definitive answers, they underscore the complexity of PLP and highlight the need for multidisciplinary approaches to unravel its molecular basis [90].

## 8. Discussions and Future Directions

Phantom limb pain remains a paradigmatic example of persistent pain occurring in the absence of ongoing peripheral tissue input. At the same time, several mechanistic interpretations remain contested. The relative contribution of peripheral versus central drivers, the causal significance of cortical reorganization, and the persistence of neuroimmune activation beyond the acute post-amputation phase continue to be debated. These uncertainties highlight the provisional nature of current explanatory models. The literature reviewed here supports the view that PLP cannot be explained by a single anatomical locus or biological process. Instead, it reflects interacting changes across peripheral nerves, spinal cord circuits, and supraspinal networks, further shaped by immune-related and long-term regulatory influences. Importantly, the relative contribution of these processes appears to vary across individuals and over time, contributing to the marked heterogeneity observed among amputees. Early neuroimaging studies provided initial evidence for central nervous system involvement in PLP. Flor et al. (1995) reported an association between alterations in somatosensory cortical representations and the presence of phantom pain, introducing the concept that deafferentation-related brain changes may influence pain perception [91]. Subsequent work by Karl et al. (2001) demonstrated that such cortical reorganization is not uniform among amputees, with differences observed between individuals with and without PLP [71]. These findings suggested that central adaptations relate to pain presence rather than amputation per se. Flor and Diers (2009) later integrated these observations into a broader framework of central nervous system plasticity in chronic pain, while emphasizing the variability of cortical changes and the limitations of direct correlations with pain intensity [92].

Parallel experimental work strengthened the biological plausibility of spinal and immune-related contributions to persistent pain following nerve injury. Tsuda et al. (2005) demonstrated activation of spinal microglia in rodent models of peripheral nerve injury, providing key evidence for neuron–glia interactions in pain amplification [85]. Scholz and Woolf (2007) subsequently synthesized molecular and cellular changes observed after nerve injury, offering a conceptual framework that has been widely extrapolated to post-amputation pain [34]. Costigan et al. (2009) further emphasized the role of immune-related signaling and glial activation in maintaining altered nociceptive processing, reinforcing the relevance of neuroimmune interactions in chronic pain states [18]. Conceptual advances helped unify these findings across different levels of the nervous system. Woolf (2011) formalized the concept of central sensitization, describing increased responsiveness within central pain pathways following injury [35]. Apkarian et al. (2011) extended this perspective by proposing that chronic pain reflects alterations in distributed brain networks rather than localized sensory abnormalities [12].

Additional regulatory layers were introduced by studies examining long-term control of gene expression. Doehring et al. (2013) reported epigenetic alterations in neuropathic pain populations, providing early translational support for stable regulatory processes contributing to pain persistence [93]. Denk et al. (2014) reviewed immune–neural interactions in chronic pain, underscoring how inflammatory signaling can influence neuronal function across peripheral and central sites [94]. An integrative translational perspective was articulated by Kuner and Flor (2016), who emphasized maladaptive plasticity arising from interactions among peripheral nerve injury, spinal processing, and supraspinal network changes [64]. More recent work has continued to refine this view. Schone et al. (2022) critically re-evaluated prevailing concepts of PLP, emphasizing unresolved questions and cautioning against oversimplified explanatory models [95].

Recent clinical studies have further highlighted inter-individual heterogeneity. Ortega-Márquez et al. (2024) examined PLP and phantom sensations in amputees, reporting associations between sensorimotor characteristics and pain variability [96]. Sparling et al. (2024) linked cortical organization to clinical features of PLP, contributing to efforts to bridge systems-level observations with patient-relevant outcomes [97]. Finally, Langeveld et al. (2024) provided important epidemiological context through a systematic review and meta-analysis demonstrating the high prevalence of PLP following dysvascular lower limb amputation, reinforcing its clinical significance [98]. Despite substantial progress, several limitations constrain interpretation of the existing literature, as summarized in Table 2. Much of the evidence remains indirect, relying on extrapolation from neuropathic pain models or correlational human data. Direct molecular investigations in amputee populations are limited, and temporal relationships between early post-amputation changes and long-term pain persistence remain poorly defined.

Future research should therefore prioritize longitudinal and integrative approaches capable of linking biological changes to clinical trajectories. Studies combining molecular profiling with neuroimaging, neurophysiological, and clinical assessments may help clarify how early post-amputation processes evolve into persistent pain and why susceptibility varies across individuals. Such approaches are likely to be essential for advancing understanding of PLP and for informing more targeted translational research strategies.

## 9. Clinical Implications and Therapeutic Perspectives

Although this review primarily addresses the molecular and cellular basis of phantom limb pain, these insights may have implications for clinical understanding and management. PLP remains difficult to treat, with highly variable responses to available interventions, and a clearer appreciation of its underlying biological complexity may help contextualize this therapeutic heterogeneity rather than immediately inform specific treatment choices [99].

The diversity of processes involved in PLP suggests that no single intervention is likely to be universally effective. Peripheral nerve injury, spinal cord plasticity, supraspinal network alterations, and immune-related processes may contribute to pain generation and persistence to different extents across individuals and disease stages. From a clinical perspective, this variability highlights the importance of viewing PLP as a dynamic and heterogeneous condition rather than a uniform clinical entity. Peripheral alterations following amputation, including changes in nociceptor excitability and local inflammatory signaling, provide a biological rationale for early attention to residual limb pain and tissue inflammation. While evidence supporting sustained peripheral modulation in established PLP is limited, early post-amputation management may influence subsequent central adaptations. However, the extent to which such approaches modify long-term phantom pain remains uncertain and requires further study [1].

At the spinal level, changes associated with central sensitization and altered neuron–glia interactions may help explain the partial and inconsistent efficacy of treatments targeting excitatory neurotransmission or inhibitory control. Pharmacological strategies that influence synaptic transmission or inflammatory signaling have shown mixed results in neuropathic pain populations, and their relevance to PLP should be interpreted cautiously. The variability observed in clinical trials likely reflects differences in underlying biological states rather than uniform treatment failure. Supraspinal network alterations may also have clinical relevance, particularly in relation to interventions that engage sensorimotor integration or cortical processing. Non-invasive neuromodulatory and sensory-based approaches have been explored in PLP with inconsistent outcomes. From a biological standpoint, such variability may be influenced by differences in the degree and nature of network-level adaptations across patients [35].

Immune-related and long-term regulatory processes add further complexity to therapeutic translation. Although these pathways have been implicated in chronic pain more broadly, current clinical strategies do not directly address persistent immune signaling or long-lasting regulation of gene expression. At present, their relevance lies primarily in shaping future research directions rather than informing immediate therapeutic decisions. Overall, insights into the biological processes associated with PLP underscore the challenges of developing uniform treatment strategies. Rather than supporting specific clinical recommendations, existing evidence suggests that treatment resistance and variability are intrinsic features of a condition driven by distributed and interacting processes. Progress toward more effective management will likely depend on translational approaches that link biological characteristics to clinical outcomes [13].

## 10. Conclusions

Phantom limb pain remains a complex and incompletely understood consequence of limb amputation, arising from interactions across multiple levels of the nervous system rather than from a single anatomical site or mechanism. Evidence suggests that peripheral nerve injury-related changes, spinal adaptations, and supraspinal network alterations may each contribute to pain development and persistence, with their relative influence varying across individuals and over time. However, direct evidence specific to PLP remains limited, and the heterogeneity of clinical presentations complicates causal inference and therapeutic translation. Methodological constraints and variability in post-amputation trajectories further warrant cautious interpretation of existing findings. Future research should adopt integrative, longitudinal approaches that link molecular and cellular processes to neurophysiological, imaging, and clinical outcomes, with the goal of clarifying mechanisms underlying persistent pain and informing more individualized management strategies.

## Figures and Tables

**Table 1:** Biological processes involved in phantom limb pain (PLP) across different levels of nervous system.

Level of the Nervous System	Principal Cell Types Involved	Biological Processes	Key Molecular and Cellular Mediators	Functional Relevance for PLP
Peripheral nervous system [20,21,22,28]	Primary sensory neurons (nociceptors), Schwann cells, immune cells	Altered neuronal excitability, ectopic activity, inflammatory signaling	Voltage-gated sodium channels, neurotrophic factors, inflammatory cytokines	Generation of abnormal afferent input following nerve injury, potentially influencing early pain signaling
Residual limb/neuroma [31,32,33]	Injured axons, Schwann cells, fibroblasts	Axonal sprouting, disorganized regeneration, spontaneous firing	Growth-associated proteins, ion channels, extracellular matrix components	Sustained aberrant signaling from the amputation site that may contribute to persistent pain
Spinal cord (dorsal horn) [35,36,37,38,85]	Projection neurons, interneurons, microglia, astrocytes	Central sensitization, reduced inhibitory control, neuron–glia interactions	Glutamatergic receptors, pro-inflammatory mediators, glial signaling molecules	Amplification and persistence of nociceptive signaling within spinal circuits
Ascending pain pathways [37,38]	Spinal projection neurons, thalamic neurons	Enhanced signal transmission, altered integration	Neurotransmitter release systems, synaptic plasticity regulators	Facilitation of persistent pain-related signaling to supraspinal centers
Supraspinal cortical regions [64,71,91]	Cortical neurons (somatosensory, motor, associative areas)	Network reorganization, altered sensorimotor integration	Activity-dependent signaling pathways, synaptic regulators	Maintenance of pain-related representations in the absence of peripheral input
Subcortical and limbic regions [79,82]	Thalamic neurons, limbic system neurons	Altered affective and cognitive pain processing	Neuromodulatory systems, synaptic plasticity factors	Contribution to the emotional and perceptual dimensions of PLP
Neuroimmune interfaces [13,85]	Microglia, astrocytes, peripheral immune cells	Immune-mediated modulation of neural function	Cytokines, chemokines, immune signaling pathways	Sustained modulation of neuronal excitability across multiple nervous system levels
Long-term regulatory processes [83,87]	Neurons and glial cells	Persistent regulation of gene expression	Epigenetic modulators, transcriptional regulators	Stabilization of long-lasting pain-related changes following transient injury

**Table 2:** Chronological key studies in phantom limb pain (PLP) research.

Year	Author	Study Type	Population/Model	Main Focus	Relevance for the Present Review
1995	Flor et al. [91]	Clinical neuroimaging	Upper and lower limb amputees	Cortical reorganization	First evidence linking altered somatosensory representations with PLP
2001	Karl et al. [71]	Clinical neuroimaging	Amputees with vs. without PLP	Sensorimotor cortex plasticity	Demonstrated that cortical reorganization differs according to pain presence
2005	Tsuda et al. [85]	Experimental	Rodent nerve injury model	Spinal microglial activation	Provided early evidence for neuron–glia interactions in pain amplification
2007	Scholtz et al. [34]	Review	Animal nerve injury models	Neuropathic pain mechanisms	Described molecular and cellular changes after nerve injury relevant to post-amputation pain
2009	Costigan et al. [18]	Review	Animal models	Neuroimmune signaling	Highlighted immune and glial contributions to pain persistence
2009	Flor et al. [92]	Review	Human studies	Central nervous system plasticity	Integrated imaging and clinical findings in PLP
2011	Woolf et al. [35]	Review	Experimental and clinical pain	Central sensitization	Defined sensitization as a core process in chronic pain
2011	Apkarian et al. [12]	Review	Chronic pain patients	Brain network alterations	Proposed chronic pain as a distributed network disorder
2013	Doehring et al. [93]	Clinical study	Neuropathic pain patients	Epigenetic regulation	Provided early clinical evidence for epigenetic involvement in chronic pain
2014	Denk et al. [94]	Review	Animal models	Immune–neural interactions	Reviewed immune modulation of neural excitability in chronic pain
2016	Kuner et al. [64]	Review	Human and animal studies	Maladaptive plasticity	Integrated peripheral, spinal, and supraspinal processes in persistent pain
2022	Schone et al. [95]	Review	PLP literature	Conceptual framework	Re-evaluated current models and unresolved questions in PLP
2024	Ortega-Marques et al. [96]	Clinical study	amputees	PLP	Linked sensorimotor features to pain variability
2024	Sparling et al. [97]	Clinical neuroimaging	Amputees	Cortical organization	Bridged cortical findings with clinical pain features
2024	Langeveld et al. [98]	Systematic review and meta-analysis	Lower limb amputees	PLP prevalence	Demonstrated high prevalence of PLP post-amputation

## Data Availability

No new data were created or analyzed in this study.
